# Stable and catalytically active iron porphyrin-based porous organic polymer: Activity as both a redox and Lewis acid catalyst

**DOI:** 10.1038/srep10621

**Published:** 2015-07-16

**Authors:** Ali R. Oveisi, Kainan Zhang, Ahmad Khorramabadi-zad, Omar K. Farha, Joseph T. Hupp

**Affiliations:** 1 Department of Chemistry, Northwestern University, 2145 Sheridan Road, Evanston, Illinois 60208, USA; 2 Faculty of Chemistry, Bu-Ali Sina University, Hamedan 65174, Iran

## Abstract

A new porphyrin-based porous organic polymer (POP) with BET surface area ranging from 780 to 880 m^2^/g was synthesized in free-base form via the reaction of meso-*tetrakis*(pentafluorophenyl) porphyrin and a rigid trigonal building block, hexahydroxytriphenylene. The material was then metallated with Fe(III) imparting activity for Lewis acid catalysis (regioselective methanolysis ring-opening of styrene oxide), oxidative cyclization catalysis (conversion of bis(2-hydroxy-1-naphthyl)methanes to the corresponding spirodienone), and a tandem catalytic processes: an *in situ* oxidation-cyclic aminal formation-oxidation sequence, which selectively converts benzyl alcohol to 2-phenyl-quinazolin-4(*3*H)-one. Notably, the catalyst is readily recoverable and reusable, with little loss in catalytic activity.

Soft microporous materials, such as metal-organic frameworks (MOFs)^1^,^2^,^3^,^4^, covalent organic frameworks^5^,^6^,^7^,^8^,^9^, polymers of intrinsic microporosity (PIMs)^10^,^11^, conjugated microporous polymers^12^,^13^,^14^, and porous organic polymers (POPs)^15^,^16^,^17^,^18^ are increasingly popular as both scaffolds and structural motifs for immobilizing and thereby heterogenizing homogeneous catalysts. POPs are highly cross-linked amorphous polymers and are typically characterized by ample molecular-scale porosity (i.e., microporosity; pore diameters ranging from a few angstroms and up to about 20 Å). The porosity may serve to: a) increase catalytic activity by increasing the number of reactant accessible catalyst sites, b) adsorb reactant molecules, thereby pre-concentrating them and boosting catalytic reaction rates, c) push multi-step reactions to completion by facilitating multiple encounters of reactive species, including intermediates, with nearby catalysts, d) constrain catalyst movement and thereby prevent catalyst deactivation via destructive encounters with other catalysts, and/or e) create micro-environments that usefully delimit reaction pathways and product distributions. These materials are typically prepared using complementary pairs of organic building blocks, A and B, via various bond formation reactions, thereby yielding materials comprising mainly A-B linkages, rather than A-A or B-B linkages^11^,^19^,^20^,^21^,^22^. Like many other schemes for catalyst heterogenization, and independent of polymer porosity, POP-based immobilization of catalysts can greatly simplify catalyst removal from completed reactions, as well as facilitate catalyst recycling^16^,^22^. POP incorporation of partially coordinated metal atoms or ions^16^,^22^, either at the building-block stage or subsequent polymerization, can create sites for pre-concentration and/or activation substrates molecules^15^,^17^,^23^. Finally, because they are assembled via covalent bond formation rather than by formation of typically weaker coordination bonds or hydrogen bonds, POPs routinely display thermal and chemical stabilities exceeding those of most MOFs and related compounds^10^,^15^,^16^,^23^,^24^,^25^,^26^,^27^,^28^, and on par with the current best-in-class for stable MOF compounds^29^,^30^.

Fe(porphyrin) units are ubiquitous in biology, most notably as O_2_ binding centers in heme proteins and as defining cofactors for the cytochrome P450 family of enzymes, which are responsible for many catalytic oxidation reactions^31^. Outside of protein environments, which serve, in part, to protect cofactors from unproductive encounters, iron porphyrins typically quickly degrade if used as oxidation catalysts. Degradation is most often bimolecular, with pairs of iron porphyrins forming catalytically inactive oxo-bridged dimers or porphyrin rings being mistaken for target substrates by other iron porphyrins. By formulating metalloporphyrin as microporous polymers, we anticipated that the cofactor-protective features of protein pockets could be mimicked, albeit by much different chemistry, to protect the porphyrins.

Although, a number of reports have explored metalloporphyrin-based polymers^7^,^9^,^10^,^15^,^17^,^32^,^33^, examples of porous metalloporphyrin polymers and their use in chemical catalysis are rare^9^,^15^,^16^,^22^. We reasoned that an additional example would perhaps yield different polymer pore or aperture sizes, different surface areas, and/or different pore volumes than found thus far for metalloporphyrin-based POPs. We also were interested in examining further the potential catalytic utility of Fe(porphyrin) POPs for three specific oxidative and/or Lewis-acid-activated transformations, as outlined briefly below.

Ring-opening of epoxides with alcohols provide a synthetically significant route to *β*-alkoxyalcohols^34^ which are an important class of organic molecules^35^. Since alcohols are poor nucleophiles, syntheses of *β*-alkoxyalcohols usually require basic or acidic conditions; unfortunately, these conditions tend to yield mixtures regioisomers and also oligomers^36^,^37^. Herein, we report that the regioselective alcoholysis of a representative epoxide has been POP-catalyzed to afford a *β*-alkoxyalcohol^34^,^38^,^39^,^40^,^41^,^42^,^43^,^44^,^45^,^46^.

Spirodienones are an attractive class of molecules obtainable by oxidative cyclization of calix[n]arenes (Fig. 1)^47^. Several reagents have been used to oxidize bis(2-hydroxy-1-naphthyl)methanes as a subunit of calix[n]arene to the corresponding spirodienones. These include phenyltrimethylammonium tribromide (PTMATB)^47^, 2,3-dichloro-5,6-dicyano-1,4-benzoquinone (DDQ)^48^, *N*-chloro reagents^49^, Ph_3_Bi^50^ and nanoparticle-supported TEMPO^51^ (2,2,6,6-tetramethylpiperidine-oxyl).

Quinazolinone derivatives^52^,^53^,^54^ have been intensively studied for their various biological and pharmacological properties such as anticancer activities and inhibiting of the epidermal growth factor (EGF) receptors of tyrosine kinase. Very recently, iridium^55^, ruthenium^56^ and I_2_/DMSO^57^ have been used for oxidative cyclization of primary alcohols with *o*-aminobenzamides to afford quinazolinones. Nevertheless, it would be desirable to catalyze their formation with less expensive catalysts and less toxic oxidants.

## Results

### POP synthesis and characterization

**Figure 2:**
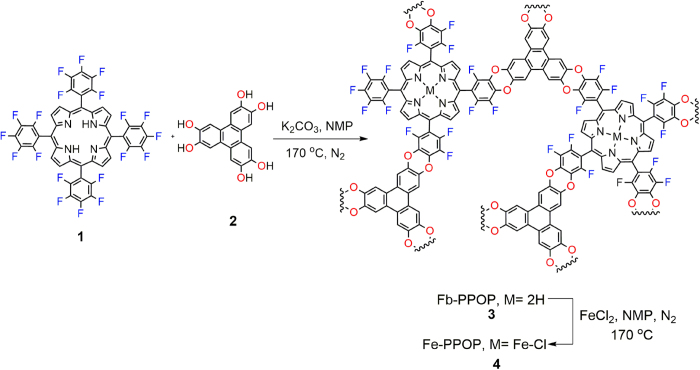
Synthesis of Fb-PPOP (**3**) and Fe-PPOP (**4**).

A free-base porphyrin POP (Fb-PPOP **3**) was prepared (by a nucleophilic substitution reaction between a tetra(pentafluoro-phenyl)porphyrin (monomer **1**) and hexahydroxytriphenylene (monomer **2**) using the conditions indicated in Fig. 2; see Supplementary Information (SI) for details (Related chemistry has been successfully used by McKeown and coworkers to assemble PIMS^10^).

Formation of ether linkages in **3** was supported by FTIR spectroscopy with the appearance of new stretching bands at 1245 and 1005 cm^−1^ (Fig. S1 in SI). Also consistent with ether formation, is the observation for **3** significantly absorption than for monomer **2** in the region between 3,000-3,500 cm^−1^ associated with O–H stretches (Fig. S1 in SI). This polymer proved insoluble in all solvents examined. Thermal gravimetric analysis measurements in air (not shown) indicated polymer degradation, as evidenced by substantial loss of mass, beginning at about 500 °C.

**Figure 3:**
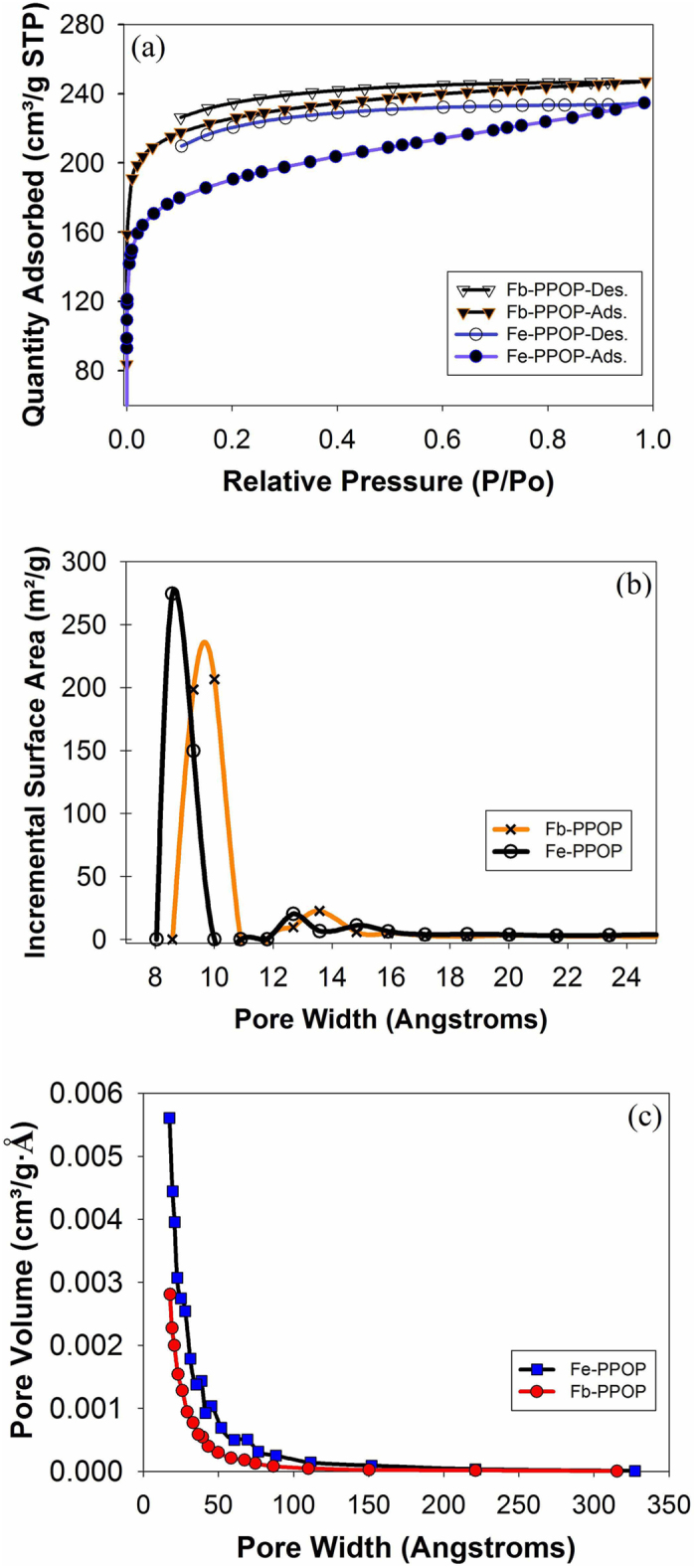
**a**) N_2_ isotherms of Fb-PPOP and Fe-PPOPs. **b**) BET-derived pore size distribution plots, and **c**) BJH adsorption pore size distributions for Fb-PPOP and Fe-PPOP. Each plot include the isotherms for the Fb-PPOP and the subsequently metallated Fb-PPOP (Fe-PPOP). BET surface area: Fb-PPOP: 877 m^2^/g; Fe-PPOP: 760 m^2^/g.

The permanent porosity of Fb-PPOP was measured via cryogenic adsorption of N_2_ which indicated to be quite porous with BET surface area of 780-880 m^2^/g (Fig. 3a). Metallation of Fb-PPOP with FeCl_2•_4H_2_O in (*N*-Methyl-2-pyrrolidone) NMP at 170 °C produced Fe-PPOP **4** (Fig. 2). The FT-IR analysis of this metallated Fb-PPOP showed a decrease in the intensity of the N-H stretching at 3318 cm^−1^ and the appearance of a new vibration band at 943 cm^−1^ which could be related to N-Fe coordinations (Fe-PPOP **4**, Fig. S2 in SI). X-ray powder diffraction analysis of the as-synthesized Fe-PPOP **4** revealed no diffraction, implying that **4** is amorphous and also suggesting that crystalline metal nanoparticles are not formed during the metallation (Fig. S8, SI). ICP-OES (inductively coupled plasma atomic emission spectroscopy) analysis revealed Fe-PPOP to contain 3.5 wt% Fe (theoretical metallation _=_ 4.1 wt%) (Table S3 in SI). The Fe metallated PPOP is porous (0.36 cm^3^/g total pore volume and 760 m^2^/g surface area with micropores of diameter ca. 9, 13, and 15 Å in Fig. 3).

**Figure 4:**
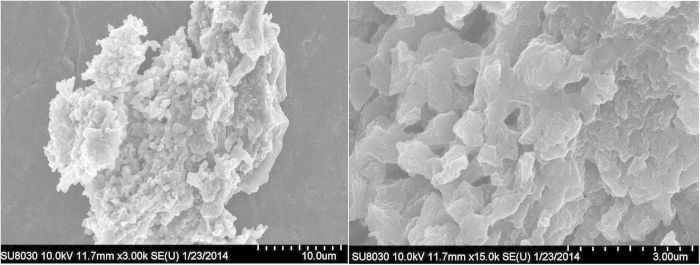
SEM images of Fe-PPOP.

N_2_ adsorption isotherm showed that BET surface area and microporous number for Fe-PPOP are slightly lower than that of Fb-PPOP (Fig. 3). The stability of the metallated PPOP was shown up to 500 °C. SEM images of Fe-PPOP show agglomerates of particles ranging in size from about 300 to 700 nm, see Fig. 4.

### POP Catalytic Activity

**Figure 5:**
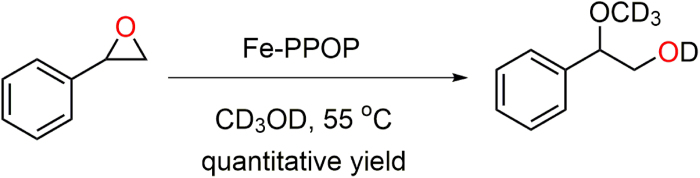
The regioselective ring opening of styrene oxide catalyzed by Fe-PPOP.

The Fe-PPOP as used as a Lewis acid catalyst for methanolysis of ring-opening of styrene oxide. At very low catalyst loading (Fe-PPOP= 10 mg equals to 0.006 mmol Fe, 1.2 mol% Fe), Fe-PPOP regioselectively (attack at the benzylic position) converts styrene oxide to *β*-methoxyalcohol within 24 h in CD_3_OD at 55 °C (Fig. 5, see also Table S1 in SI).

**Figure 6:**
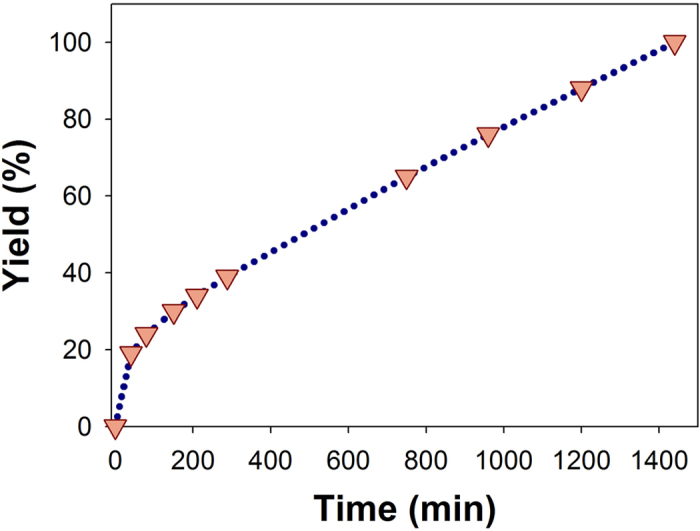
*β*-methoxyalcohol formation profile for the regieoselective ring opening of styrene oxide in the presence of Fe-PPOP catalyst (0.006 mmol Fe).

The conversion profile for styrene oxide is shown in Fig. 6. This methanolysis reaction was optimized to give the *β*-methoxyalcohol in quantitative yield (Table S1, in SI).

At the end of the reaction, inductively coupled plasma (ICP) analysis of the reaction mixture filtrate revealed no Fe leaching, consistent with the anticipated heterogeneous nature of the catalyst. Additionally, the catalyst was reused without a significant decrease in its efficiency (see Table S1 in SI). The above reaction confirmed that Fe-PPOP could act as a Lewis acid catalyst to activate epoxides.

**Figure 7:**
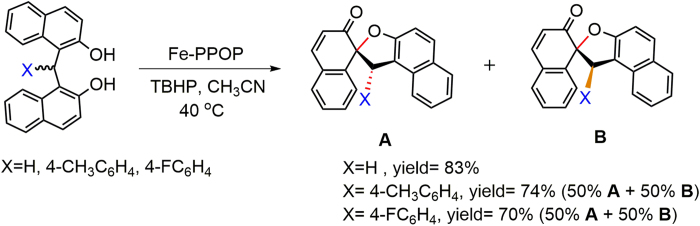
Oxidative cyclization of bis(2-hydroxy-1-naphthyl)methanes to the corresponding spirodienones by Fe-PPOP.

We next evaluated Fe-PPOP as a catalyst for oxidative cyclization of bis(2-hydroxy-1-naphthyl)methanes by TBHP (*tert*-butyl hydroperoxide) (Fig. 7). Screening experiments in various solvents, including acetonitrile, dichloromethane and ethanol, pointed showed to acetonitrile as the most suitable reaction medium (Table S2 in SI). The optimized condition led to synthesis of the corresponding oxidation products, spirodienones (Table S2, SI). When Fe-PPOP recycled, its activity slightly reduced after three cycles (Table S2, SI). ICP-OES analysis showed little change in Fe content following recycling (Table S3, SI).

**Figure 8:**
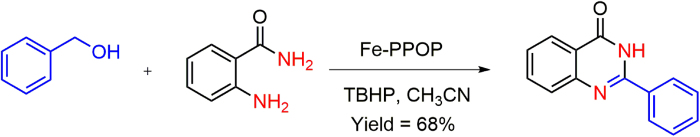
The tandem catalytic conversion of benzyl alcohol to 2-phenyl-quinazolin-4(*3*H)-one by Fe-PPOP.

This catalyst was also used for a tandem catalytic process: an *in situ* oxidation-cyclic aminal formation-oxidation sequence. As indicated in Fig. 8, benzyl alcohol reacts with *o-*aminobenzamide to yield 2-phenyl-quinazolin-4(*3*H)-one, with Fe-PPOP functioning as both an oxidation catalyst and Lewis which shows catalytically activity as both Lewis acid and redox iron site (Table S4 in SI).

## Discussion

In summary, we have developed the synthesis of a stable and catalytically active iron porphyrin-based porous organic polymer featuring a good surface area. Both the free-base and metallated versions of the porphyrin-based POP can be readily synthesized. This reusable catalyst can act as both a Lewis acid and redox-active site. FePPOP catalyzes: a) the regioselective methanolysis ring opening of styrene oxide, b) oxidative cyclization of bis(2-hydroxy-1-naphthyl)methanes to the corresponding spirodienone, and c) a tandem process (an *in situ* oxidation-cyclic aminal formation-oxidation sequence) which converts benzyl alcohol to 2-phenyl-quinazolin-4(*3*H)-one.

## Methods

### Preparation of Fb-PPOP

In a nitrogen glove box, a 20 mL microwave vial (capacity designates the amount of solution that can be safely loaded) equipped with a magnetic stir bar was charged with *meso-tetrakis(pentafluorophenyl)porphyrin* (100 mg, 0.103 mmol), *2,3,6,7,10,11-hexahydroxytriphenylene* (50.2 mg, 0.155 mmol) and K_2_CO_3_ (385 mg, 2.76 mmol). Anhydrous NMP (3-4 mL) was then added to the resulting solution and the microwave vial was sealed with a crimp cap. The vial was removed from the glove box and placed into a 170 °C oil bath where the reaction mixture was allowed to stir for 3 h. The solution thickened and precipitates were observed after about 10 min; after 30 min, significant gelation could be observed. After 3 h, the vial was removed from the oil bath and cooled down slightly, the crimp cap was removed and methanol (15 mL) was then added to the mixture. Then the solid was washed with DMF, dichloromethane, acetone, methanol and water. The solid was purified by Soxhlet extraction in methanol/ water for 12 h, refluxed in acetone (2 × 1 h) and then filtered. Removal of solvent under vacuum at 120 °C gave a dark purple solid **3** (133 mg, 99% yield). Supplementary information [materials and characterization (CHN analysis, N_2_ adsorption, BET surface area, SEM images, PXRD pattern and FTIR spectra)] accompanies this paper.

### Preparation of Fe-PPOP

In a 25-mL Schlenk flask equipped with a magnetic stir bar, Fb-PPOP **3** (50 mg) and FeCl_2_ (77 mg, 10 equiv to Fb-PPOP) were placed under N_2_ and dry NMP (10 mL) was added by syringe. The reaction mixture was heated at 170 °C for 24 h before being cooled to room temperature. The reaction flask was opened to air, HCl (1 M) and water (50:50) were added, and the reaction mixture was stirred for 5 h. The solid product was filtered and washed with water (50 mL), acetone (30 ml), dichloromethane (20 ml), tetrahydrofuran (10 mL), and then purified by Soxhlet extraction in methanol for 10 h. Drying under vacuum at 120 °C gave a dark solid **4** (52 mg). Supplementary information including characterization date (CHN analysis, N_2_ adsorption, BET surface area, SEM images, ICP-OES analysis, PXRD pattern and FTIR spectra) accompanies this paper.

### Methanolysis of styrene oxide catalyzed by Fe-PPOP

Styrene oxide (57 μL, 0.5 mmol), deuterated methanol (0.5 ml) and Fe-PPOP (10 mg, equivalent to 0.006 mmol Fe) were added to a 1 mL micro-centrifuge tube and then sealed. Then the vial was placed in a thermo-shaker at 55 °C for 24 h. NMR spectra were recorded at different time intervals. Then at the end of the reaction, the vial was cooled to room temperature and opened. After catalyst separation by centrifugation, a small aliquot of the supernatant reaction mixture was taken to be analyzed by ^1^H NMR to calculate the conversion, regioselectivity and the yield of the reaction (see Supplementary information).

### General Procedure for oxidative cyclization of bis(2-hydroxy-1-naphthyl)methanes to the corresponding spirodienone using of Fe-PPOP

To a round-bottom flask (25 ml), a mixture of bis(2-hydroxy-1-naphthyl)methane (1 mmol), Fe-PPOP (15 mg, equivalent to 0.009 mmol of Fe) was poured in acetonitrile (10-15 ml). Then TBHP (3 mmol) was added, stirred and maintained at 40 °C. The progress of the reaction was followed by TLC. After completion of the reaction, the catalyst (Fe-PPOP) was separated by centrifuge, the excess solvent concentrated by evaporation and the crude mixture was purified by column chromatography (ethyl acetate:*n*-hexane, 2:10) to obtain the pure product (see Supplementary information).

### Tandem catalytic synthesis of 2-phenyl-quinazolin-4(*3*H)-one through the reaction of benzyl alcohol and o-aminobenzamide using of Fe-PPOP

To a mixture of benzyl alcohol (1.5 mmol), *o*-aminobenzamide (0.5 mmol), Fe-PPOP (20 mg, equivalent to 0.012 mmol of Fe) in acetonitrile (3 ml), TBHP (70% in water) (2.5 mmol: 1.5 mmol, 0.5 mmol, 0.5 mmol) was added into three portions, the first was added after 10 min, second after 10 h and the third after 18 h. The reaction mixture was stirred at 60 °C for 24 h. After completion of the reaction (monitored by TLC) the mixture was cooled and concentrated in vacuo. Then the catalyst was washed with ethyl acetate and centrifuged and the supernatant solution was decanted. This process was repeated for three times. The residue was then purified by chromatography on silica gel (*n*-hexane/ethyl acetate) to afford the pure product in 68% yield (see Supplementary information).

## Additional Information

**How to cite this article**: Oveisi, A. R. *et al.* Stable and catalytically active iron porphyrin-based porous organic polymer: Activity as both a redox and Lewis acid catalyst. *Sci. Rep.* **5**, 10621; doi: 10.1038/srep10621 (2015).

## Supplementary Material

Supplementary Information

## Figures and Tables

**Figure 1:**
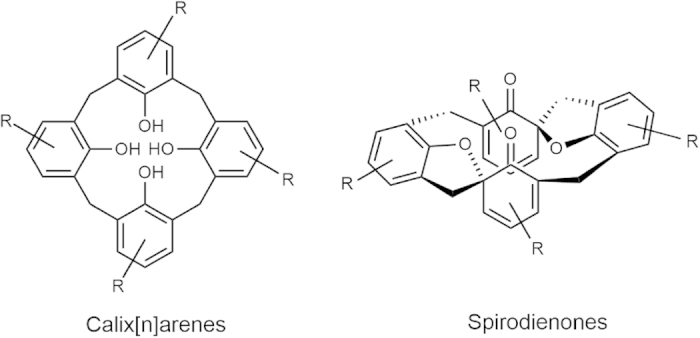
Spirodienones and calix[n]arenes.
