# Supplementary material for: Stable and catalytically active iron porphyrin-based porous organic polymer: Activity as both a redox and Lewis acid catalyst
Source: Sci Rep. 2015 Jul 16;5:10621. doi: 10.1038/srep10621 (PMC4503946; doi:10.1038/srep10621)
Supplement: Supplementary Information [file srep10621-s1.pdf]

Supplementary Information

Stable and catalytically active iron porphyrin-based porous organic polymer: Activity as both a redox and Lewis acid catalyst

Ali R. Oveisi [1,2], Kainan Zhang[1], Ahmad Khorramabadi-zad[2], Omar K. Farha*[1] and Joseph T. Hupp*[1]

[1] Department of Chemistry, Northwestern University, 2145 Sheridan Road, Evanston, Illinois 60208, USA.

[2] Faculty of Chemistry, Bu-Ali Sina University, Hamedan 65174, Iran

Corresponding authors:

E-mail Address: *j-hupp@northwestern.edu* (Joseph T. Hupp) and *o-farha@northwestern.edu* (Omar K. Farha)

Table of Contents	*Page number*
S1. General information.	*S1*
S2. General procedures and materials.	*S2*
S3. Preparation of Fb-PPOP.	*S2*
S4. FTIR spectra of Fb-PPOP.	*S4*
S5. Preparation of Fe-PPOP by post-synthesis modification.	*S5*
S6. FTIR spectra of Fe-PPOP.	*S6*
S7. N_2 isotherms of Fb-PPOP and Fe-PPOP.	*S7*
S8. Pore size distribution of Fb-PPOP and Fe-PPOP.	*S7*
S9. SEM images of Fb-PPOP.	*S8*
S10. SEM images of Fe-PPOP.	*S9*
S11. PXRD patterns of Fe-PPOP.	*S9*
S12. Methanolysis of ring opening of styrene oxide catalyzed by Fe-PPOP	*S10*
S13. General Procedure for oxidative cyclization of bis(2-hydroxy-1-naphthyl)methanes to the corresponding spirodienone using of Fe-PPOP.	*S11*
S14. ICP-OES Analysis.	*S13*
S15. Tandem catalytic synthesis of 2-phenyl-quinazolin-4(*3*H)-one through the reaction of benzyl alcohol and *o*-aminobenzamide using of Fe-PPOP.	*S13*
S16. ^{1}HNMR spectra.	*S14*
S17. References	*S19*

S1. General information.

Powder X-ray diffraction (PXRD) patterns were recorded on a Rigaku XDS 2000 diffractometer using nickel-filtered Cu Kα radiation (λ = 1.5418 Å) over a range of 5° < 2θ < 40°. Simulations were made based on the single-crystal data using the Mercury software. The powder samples were mounted on clear cellophane tape and PXRD data were collected immediately after mounting.

The powder patterns were treated for amorphous background scatter. Thermogravimetric analysis (TGA) was performed on a Mettler Toledo TGA under air and nitrogen flows and heated from room temperature to 700 °C (at 10 °C/min).

Inductively coupled plasma optical emission spectroscopy (ICP-OES) was conducted on a Varian Vista MPX ICP- OES instrument that is equipped to cover the spectral range from 175 to 785 nm. Samples (3 mg) were digested in conc. H_2SO_4:30% aq. H_2O_2 (3:1 v/v) and heated at 120 °C until the solution became clear and colorless.

Samples for scanning electron microscopy (SEM) were sputtered with a layer of Os (5-nm thickness) prior to taking images on a Hitachi S-4800 SEM with a 15.0 kV accelerating voltage. Elemental analyses were provided by Atlantic Microlab, Inc. (Norcross, GA).

All adsorption and desorption measurements were performed on a Micromeritics Tristar 3020 (N_2) system (Micromeretics, Norcross, GA) and measured at 77 K. Between 40-100 mg of samples were employed in each measurement and the data were analyzed using the ASAP 2020 instrument (Micromeretics, Norcross, GA). Before measurements, thermally activated samples were degassed for 12 h at 120 ºC under high vacuum (< 10-4 bar).

The pore size distributions were calculated from the adsorption-desorption isotherms. ^{1}H NMR spectra were recorded on Jeol 90 MHz, 400 and 500 MHz Agilent DD MR-400 system equipped with Agilent 7600 96-sample auto-sampler. ^{1}H NMR chemical shifts are reported in ppm downfield from tetramethylsilane (TMS, δ scale) using the residual solvent resonances as internal standards. Fourier Fourier-transformed infrared (FTIR) spectroscopy spectra were recorded using a Perkin Elmer Spectrum-FTIR Version 10.01.00.

S2. General procedures and materials.

Bis(2-hydroxy-1-naphthyl)methanes [(methylenbisnaphthol) and Arylbisnaphthols] were synthesized following Mironov and Hewitt's methods.[1] 2-Naphthol (Sigma-Aldrich, 99%), sodium acetate (Sigma-Aldrich, 99%), Formaldehyde solution (Sigma-Aldrich, 37 wt% in H_2O), anhydrous 1-Methyl-2-pyrrolidinone (Sigma-Aldrich, 99.5%), 5, 10, 15, 20-tetrakis (pentafluorophenyl)porphyrin (Frontier Scientific, Inc. and Sigma-Aldrich), iron *meso*-tetrakis(pentafluorophenyl)porphyrin (Frontier Scientific, Inc. or Aldrich), Iron(II) chloride tetrahydrate (Sigma-Aldrich, 99%), anhydrous K_2CO_3 (Sigma-Aldrich, 98%), styrene oxide (Sigma-Aldrich, 97%), *tert*-butyl hydroperoxide (Sigma-Aldrich, 70% in H_2O), *o*-aminobenzamide (Sigma-Aldrich, ≥ 98%), 2,3,6,7,10,11-Hexahydroxytriphenylene Hydrate (TCI, 95.0%), benzyl alcohol (Merck, for synthesis), and solvents were purchased from Sigma-Aldrich Chemicals Co. All deuterated solvents were purchased from Cambridge Isotope Laboratories or Sigma-Aldrich and used as received.

S3. Preparation of Fb-PPOP by thermal activation.

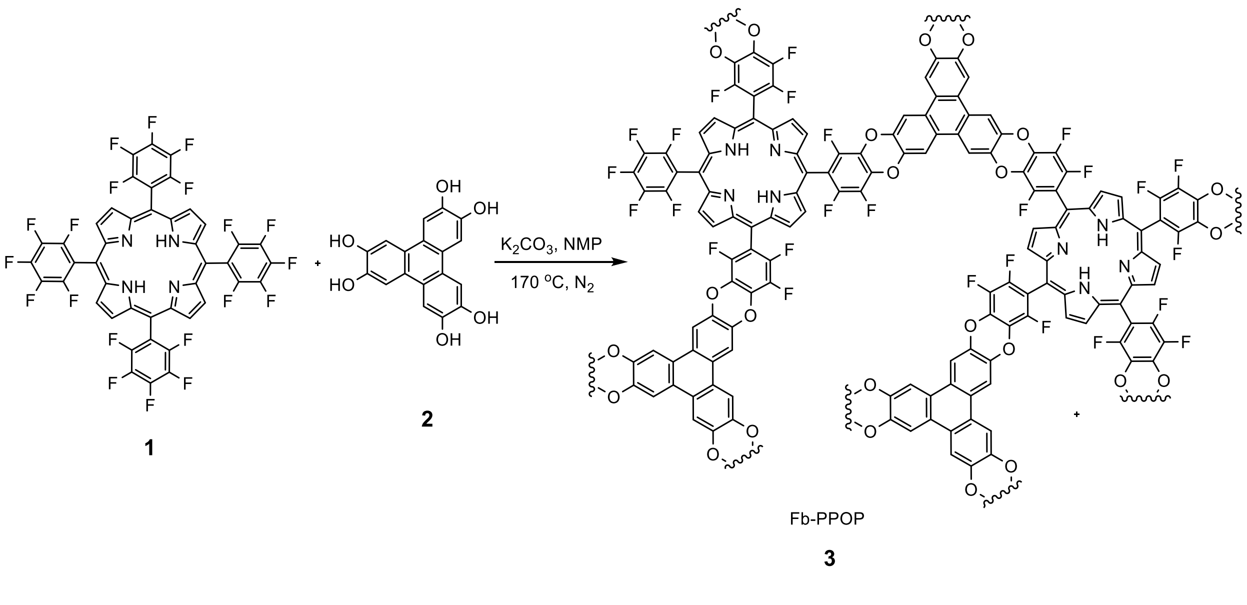

In a nitrogen glove box, a 20 mL microwave vial (capacity designates the amount of solution that can be safely loaded) equipped with a magnetic stir bar was charged with *meso-tetrakis(pentafluorophenyl)porphyrin* **1** (100 mg, 0.103 mmol), *2,3,6,7,10,11-*

hexahydroxytriphenylene **2** (50.2 mg, 0.155 mmol) and K_2CO_3 (385 mg, 2.76 mmol). Anhydrous NMP (3-4 mL) was then added to the resulting solution and the microwave vial was sealed with a crimp cap. The vial was removed from the glove box and placed into a 170 ºC oil bath where the reaction mixture was allowed to stir for 3 h. The solution thickened and precipitates were observed after about 10 min; after 30 min, significant gelation could be observed. After 3 h, the vial was removed from the oil bath and cooled down slightly, the crimp cap was removed and methanol (15 mL) was then added to the mixture. Then the solid was washed with DMF, dichloromethane, acetone, methanol and water. The solid was purified by Soxhlet extraction in methanol/water for 12 h, refluxed in acetone (2 × 1 h) and then filtered. Removal of solvent under vacuum at 120 ºC gave a dark purple solid **3** (133 mg, 99 % yield). Anal.: Calcd for $(C_{68}H_{20}F_{12}N_4O_8)_n$: C, 65.40; H, 1.61; N, 4.49. Found: C, 55.91; H, 2.19; N, 4.56.

S4. FTIR spectra of Fb-PPOP.

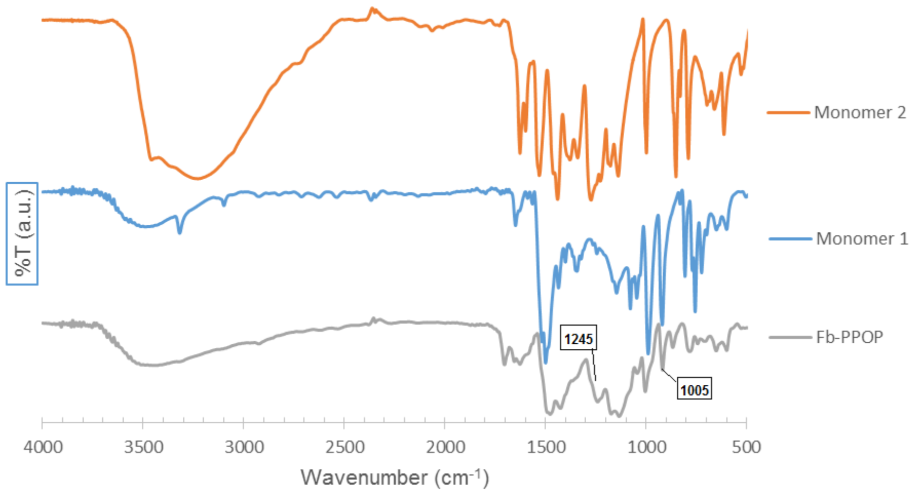

Figure S1 FTIR spectra of monomer **1** (*meso*-tetrakis(pentafluorophenyl) porphyrin), monomer **2** (2,3,6,7,10,11-Hexahydroxytriphenylene) and Fb-PPOP **3**.

S5. Preparation of Fe-PPOP by post-synthesis modification.

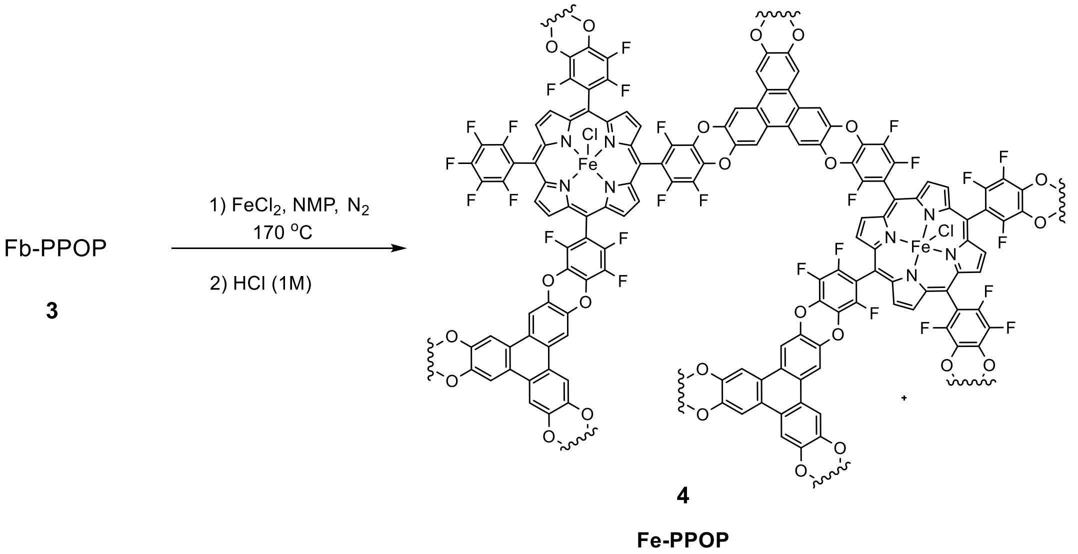

In a 25-mL Schlenk flask equipped with a magnetic stir bar, Fb-PPOP **3** (50 mg) and $FeCl_2$ (77 mg, 10 equiv to Fb-PPOP) were placed under N_2 and dry NMP (10 mL) was added by syringe. The reaction mixture was heated at 170 °C for 24 h before being cooled to room temperature. The reaction flask was opened to air, HCl (1 M) and water (50:50) were added, and the reaction mixture was stirred for 5 h. The solid product was filtered and washed with water (50 mL), acetone (30 ml), dichloromethane (20 ml), tetrahydrofuran (10 mL), and then purified by Soxhlet extraction in methanol for 10 h. Drying under vacuum at 120 °C gave a dark solid **4** (52 mg). Anal.: Calcd for $(C_{68}H_{18}ClF_{12}FeN_4O_8)_n$: C, 61.03; H, 1.36; N, 4.19. Found: C, 54.06.11; H, 1.90; N, 4.40. ICP-OES: 3.5 wt% Fe (Theoretical: 4.1 wt%Fe).

S6. FTIR spectra of Fe-PPOP.

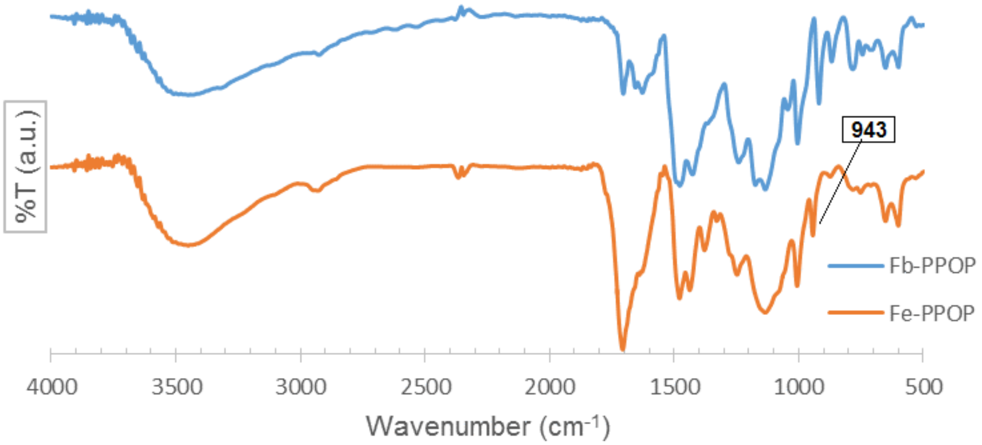

Figure S2 FTIR spectra of Fb-PPOP and Fe-PPOP.

It should be noted that metallation by post-synthesis modification under microwave radiation at 170 °C for 1 h gives only 2.9 wt% of Fe.

Also Fe-PPOP was prepared under the same protocol, with exception to the use of iron porphyrin instead of free base porphyrin scale as described for Fb-PPOP, from reaction of *iron meso-tetrakis*(*pentafluorophenyl*)*porphyrin* (90 mg, 0.0847 mmol), *hexahydroxytriphenylene* **2** (45 mg, 0.127 mmol), K_2CO_3 (210 mg, 1.52 mmol) under N_2 and dry NMP (4 ml) at 170 °C for 3 h to give dark solid (120 mg, 89 % yield). ICP-OES shows only 1.2 wt% Fe.

S7. N_2 isotherms of Fb-PPOP and Fe-PPOP.

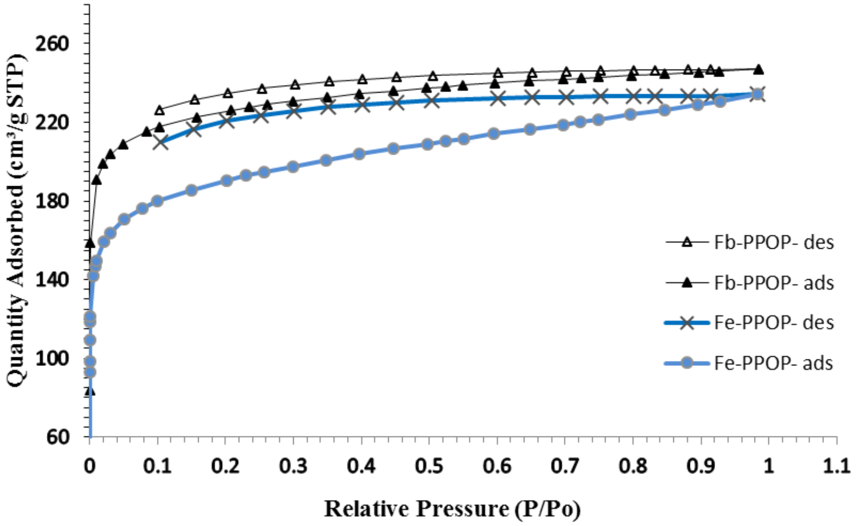

Figure S3 N_2 isotherms of Fb-PPOP and Fe-PPOPs. Each plot include the isotherms for the Fb-PPOP and the subsequently metallated PPOP. BET surface area: Fb-PPOP: 877 m^2/g; Fe-PPOP: 760 m^2/g.

S8. Pore size distribution of Fb-PPOP and Fe-PPOP.

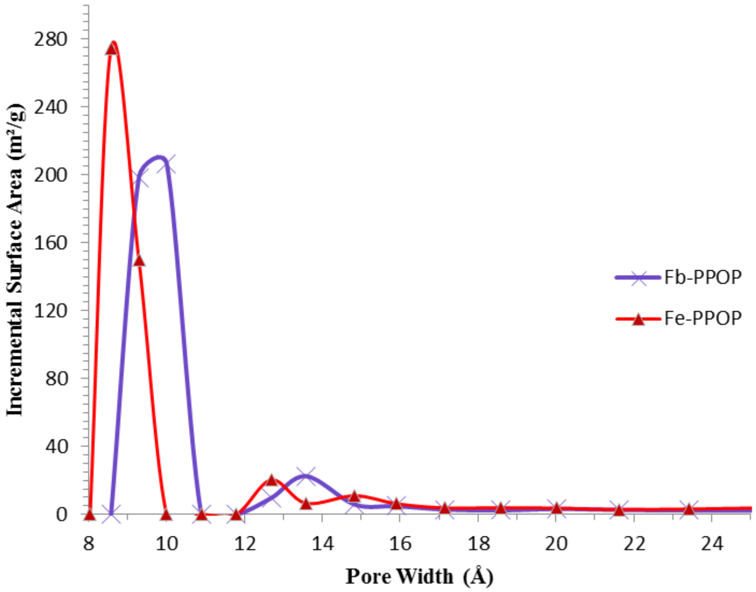

Figure S4 BET-derived pore size distribution plots for Fb-PPOP (blue cross) and Fe-PPOP (red triangle).

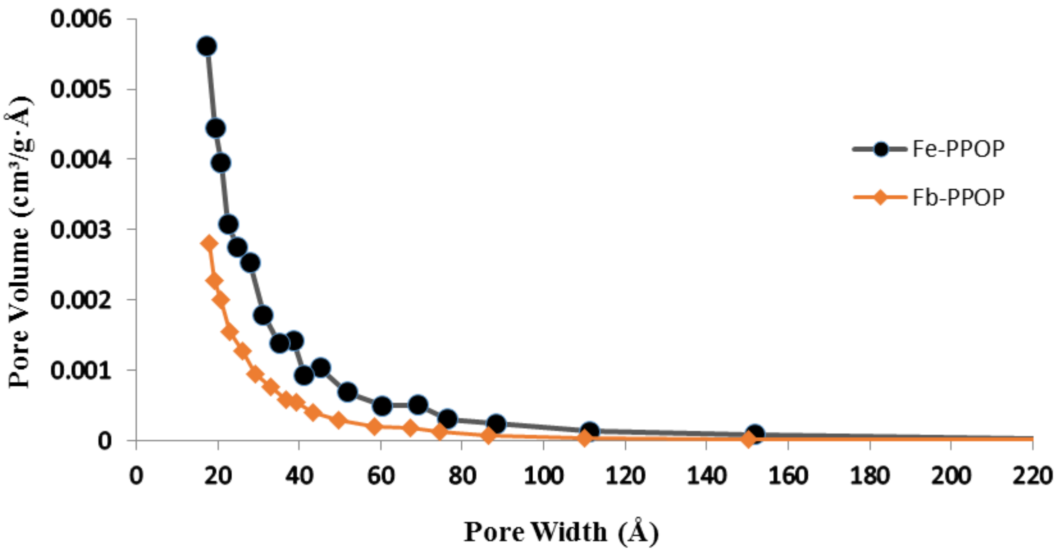

Figure S5 BJH adsorbtion pore size distributions for Fb-PPOP (orange squares) and Fe-PPOP (black circles).

S9. SEM image of Fb-PPOP.

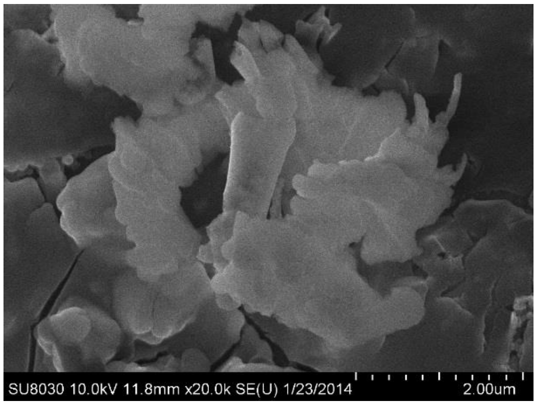

Figure S6 SEM image of Fb-PPOP.

S10. SEM images of Fb-PPOP.

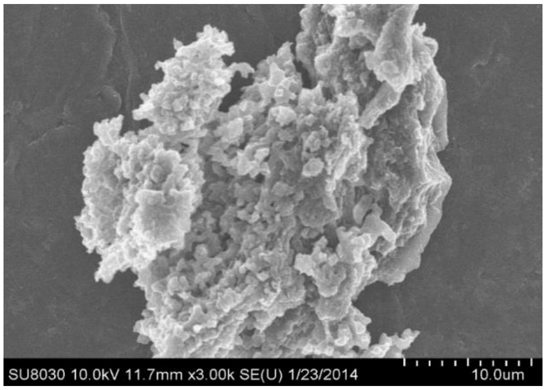

Fe-PPOP

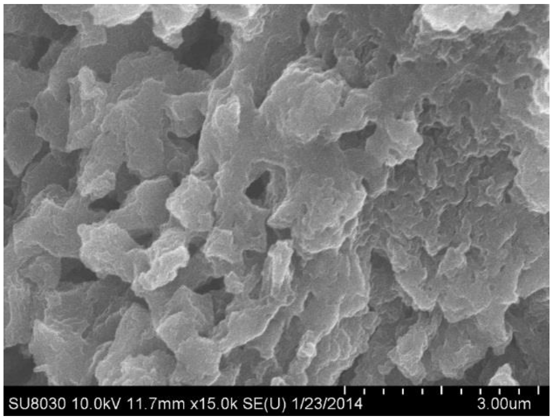

Fe-PPOP

Figure S7 SEM images of Fe-PPOP.

S11. PXRD patterns of Fe-PPOP.

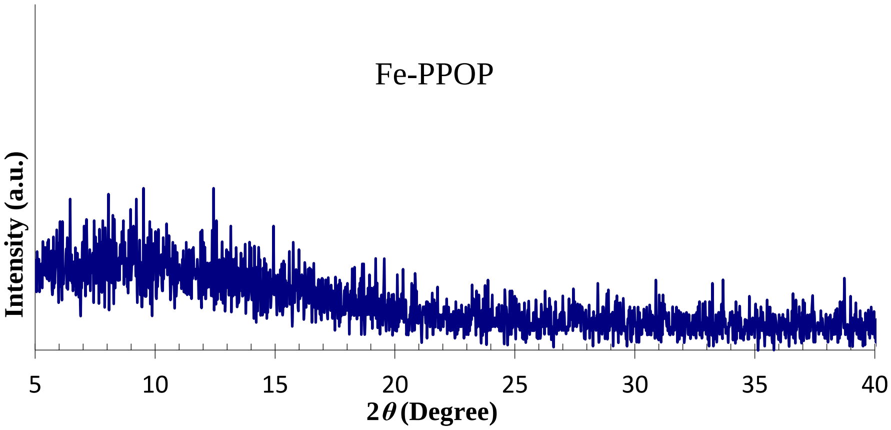

Figure S8 PXRD patterns of activated (heated under vacuum at 120 °C for 12 h) sample of **Fe-PPOP.**

S12. Methanolysis of styrene oxide catalyzed by Fe-PPOP.

Styrene oxide (57 μL, 0.5 mmol), deuterated methanol (0.5 ml) and Fe-PPOP (10 mg, equivalent to 0.006 mmol Fe) were added to a 1 mL micro-centrifuge tube and then sealed. Then the vial was placed in a thermo-shaker at 55 °C for 24 h. NMR spectra were recorded at different time intervals. Then at the end of the reaction, the vial was cooled to room temperature and opened. After catalyst separation by centrifugation, a small aliquot of the supernatant reaction mixture was taken to be analyzed by ^{1}H NMR to calculate the conversion, regioselectivity and the yield of the reaction. ^{1}H NMR (400 MHz, C_6D_6): δ 6.98–7.09 (m, 5H), 4.02 (dd, *J* = 8, 4 Hz, 1H), 3.58 (dd, *J* = 12, 8 Hz, 1H), 3.46 (dd, *J* = 12, 4 Hz, 1H).

$Fe(ClO_4)_3$[2] and $FeCl_3$[3] supported on silica gel were reported to act as heterogeneous catalysts for ring-opening of epoxides. However, leaching studies for these catalysts are not included in the reports. The reaction can just as well be catalyzed by the Fe ions that are possibly leached from the support materials, which is not uncommon for supported metal catalysts.

Table S1. Ring-opening of styrene oxide using Fe-PPOP as Catalyst.

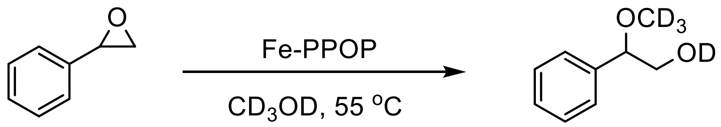

Entry	Catalyst	Time (h)	Yield (%)[d]	Selectivity (%)[d]	TON/TOF(h^{-1})[g]
1[a]	-	1		-	-
2[a]	Fe-PPOP	1	21	100	17.5/17.5
3[a]	Fe-PPOP	24	>99	100	82.5/3.43
4[b]	Fe-PPOP	24	>99	100	82.5/3.43
5[c]	Fe(BTC)	24	72[e]	95[e]	59.7/2.5
6[f]	Hf-NU-1000	52	90	100[f]	25/0.48

[a] Condition: styrene oxide (57 μL, 0.5 mmol), Fe-PPOP (10 mg, 0.006 mmol of Fe), 55˚C, CD_3OD (0.5 ml). Reaction between styrene oxide and MeOH without the catalyst at room-temperature gave no product in 12 h.

[b] Reusability of Fe-PPOP in the second run.

[c] Data derived from ref.[[1]]. Condition: Fe(BTC) (50 mg, 0.2 mmol of Fe), styrene oxide (2 mL, 16.6 mmol), methanol (5 mL) at 40 °C.

[d] Determined by ^{1}H NMR using 1, 4-dimethoxy benzene as the internal standard.

[e] Determined by GC using nitrobenzene as the external standard.

[f] Data derived from ref.[2] Condition: Hf-NU-1000 (4 mol%), Styrene oxide (0.2 mmol), CD_3OD (0.24 mL) at 55 °C.

[g] TON = turnover number (mmol of product per mmol of catalyst), TOF = turnover frequency (TON per time of reaction).

S13. General Procedure for oxidative cyclization of bis(2-hydroxy-1-naphthyl)methanes to the corresponding spirodienone using of Fe-PPOP.

To a round-bottom flask (25 ml), a mixture of bis(2-hydroxy-1-naphthyl)methane (1 mmol), Fe-PPOP (15 mg, equivalent to 0.009 mmol of Fe) was poured in acetonitrile (10-15 ml). Then TBHP (3 mmol) was added, stirred and maintained at 40 °C. The progress of the reaction was followed by TLC. After completion of the reaction, the catalyst (Fe-PPOP) was separated by centrifuge, the excess solvent concentrated by evaporation and the crude mixture was purified by column chromatography (ethyl acetate:*n*-hexane, 2:10) to obtain the pure product. All of the desired products were characterized by comparison of their physical and spectra data with those of known compounds.[1, 6]

Spiro[naphthalene-1(2H),2′(1′H)-naphtho[2,1-b]furan]2-one (Table S2, Entry 3):

Yellow solid, yield 83%; m.p: 171–172 °C. IR (ν_{max}, cm^{-1}): 1685 (C=O). ^{1}H NMR (90 MHz, $CDCl_3$): δ3.50 and 4.1 (dd, *J* = 16 Hz, 2H); 6.30 (d, *J* = 9.9 Hz, 1H), 6.90–7.99 (11H).

1′-(4-Methylphenyl)-spiro[naphthalene-1(2H),2′(1′H)-naphtho[2,1-b]furan]-2-one (Table S2, Entry 6) (Isomer **A** and **B):**

Yellow solid, yield 74%; IR (ν_{max}, cm^{-1}): 1680 (C=O). ^{1}H NMR (500 MHz, $CDCl_3$): δ2.12 and 2.27 (s, 3H), 5.18 and 5.37 (s, 1H), 5.55 and 6.28 (d, *J* = 9.99 and 9.92 Hz, 1H), 6.66–7.89 (15H).

1′-(4-Fluorophenyl)-spiro[naphthalene-1(2H),2′(1′H)-naphtho[2,1-b]furan]-2-one (Table S2, Entry 7) (Isomer **A** and **B):**

Yellow solid, yield 70%; IR (ν_{max}, cm^{-1}): 1676 (C=O). ^{1}H NMR (500 MHz, $CDCl_3$): δ5.19 and 5.38 (s, 1H), 5.57 and 6.27 (d, *J* = 9.98 Hz and *J* = 9.92 Hz, 1H); 6.26–7.9 (15H).

For the recycling experiment, the recovered catalyst was washed with acetonitrile and acetone, then centrifuged and the supernatant solution was decanted. This process was repeated for three times and then the dried catalyst was reused for the next cycle.

Table S2. Oxidative cyclization of bis(2-hydroxy-1-naphthyl)methanes to the corresponding spirodienone using of Fe-PPOP.

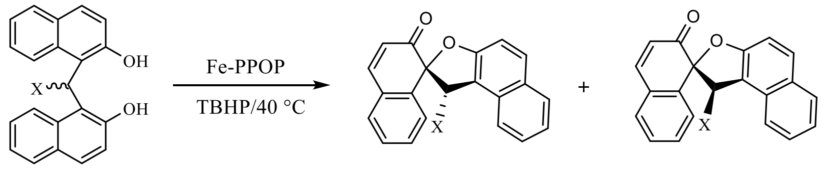

A B

X=H, 4-$CH_3C_6H_4$, 4-FC_6H_4

Entry	X	Solvent	Time (h)	Yield (%)[b]	Diastereselectivity (%)[c]		TON/TOF (h^{-1})
					**A**	**B**	
1	H	EtOH	12	40	-		44.4/3.7
2	H	CH_2Cl_2	12	67	-		74.4/6.2
3	H	CH_3CN	12	83, 74[d], 72[e]	-		92.2/7.6
4[a]	H	CH_3CN	12	20	-		-
5	H	-	12	-	-		-
6	4-$CH_3C_6H_4$	CH_3CN	11	74	50	50	82.2/7.5
7	4-FC_6H_4	CH_3CN	10	70	50	50	77.8/7.8

Reaction conditions: Bisnaphthol (1 mmol), (15 mg, 0.009 mmol of Fe), TBHP (3 mmol), solvent (10–15 mL), 40 °C.

[a] without Fe-PPOP.

[b] Total yield (isomer A + isomer B).

[c] Diastereomeric ratio. The ratio of the two diastereomers, **A** and isomer **B**, was determined by ^{1}H NMR.

[d] Reuse of Fe-PPOP in second run.

[e] Reuse of Fe-PPOP in third run, TON = 80, TOF = 6.17 (h^{-1}).

S14. ICP-OES Analysis.

Table S3. ICP-OES results for oxidative cyclization of cyclization of bis(2-hydroxy-1-naphthyl)methane by TBHP.

Name	Pristine	After catalysis
Fe-PPOP	3.5%	3.2%

S15. Tandem catalytic synthesis of 2-phenyl-quinazolin-4(*3*H)-one through the reaction of benzyl alcohol and *o*-aminobenzamide using of Fe-PPOP.

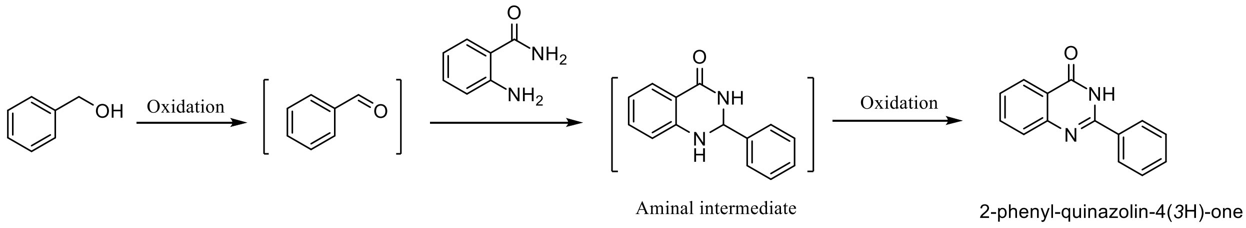

To a mixture of benzyl alcohol (1.5 mmol), *o*-aminobenzamide (0.5 mmol), Fe-PPOP (20 mg, equivalent to 0.012 mmol of Fe) in acetonitrile (3 ml), TBHP (70% in water) (2.5 mmol: 1.5 mmol, 0.5 mmol, 0.5 mmol) was added into three portions, the first was added after 10 min, second after 10 h and the third after 18 h.. The reaction mixture was stirred at 60 ^oC for 24 h. After completion of the reaction (monitored by TLC) the mixture was cooled and concentrated in vacuo. Then the catalyst was washed with ethyl acetate and centrifuged and the supernatant solution was decanted. This process was repeated for three times. The residue was then purified by chromatography on silica gel (*n*-hexane/ethyl acetate) to afford the pure product in 68% yield. The desired product was characterized by comparison of their physical and ^{1}H-NMR data with those of known compounds.[7-9] White solid, mp: 232–235 °C. ^{1}H NMR (400 MHz, DMSO-d_6) δ 12.50 (br, 1H), 8.13-8.16 (m, 3H), 7.82 (t, *J* = 7.1 Hz, 1H), 7.73 (d, *J* = 8.0 Hz, 1H), 7.49 –7.58 (m, 4H).

Table S4. Tandem catalytic synthesis of 2-phenyl-quinazolin-4(*3*H)-one through the reaction of benzyl alcohol and *o*-aminobenzamide.

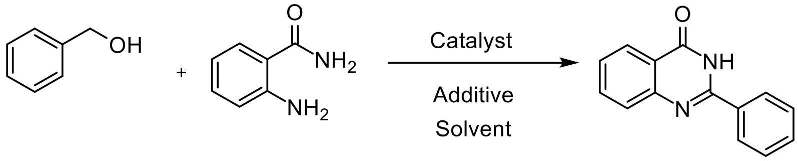

Entry	Catalyst (mol%)	Additive	Solvent	T (°C)	Time (h)	Yield (%)
1	$Ru(PPh_3)_3(CO)(H)_2$ (5 mol%)[b]	Xantphos, Crotononitrile	Toluene	110	24	72
2	$[Cp^*IrCl_2]_2$ (2.5 mol%)[c]	-	Toluene	110	36	93
3	I_2 (5 mol%)[d]	DMSO	Dimethyl carbonate	100	15	91
4[a]	Fe-PPOP (2.4 mol% of Fe)	TBHP	DMSO	60	24	60
5[a]	Fe-PPOP (2.4 mol% of Fe) (this work)[e]	TBHP	CH_3CN	60	24	68
6[a]	-	TBHP	CH_3CN	60	18	trace

[a] Condition: Benzyl alcohol (1.5 mmol), *o*-aminobenzamide (0.5 mmol), (20 mg, 0.012 mmol of Fe), TBHP (2.5 mmol), solvent (1–3 mL), 60 ºC.
[b] Date derived from ref.[3]

[c] Date derived from ref.[4]

[d] Date derived from ref.[5] It should be noted that the reaction carried out in two steps and sequence addition.

[e] TON = 28.3, TOF = 1.2 (h^{-1}).

S16. ^{1}HNMR spectra

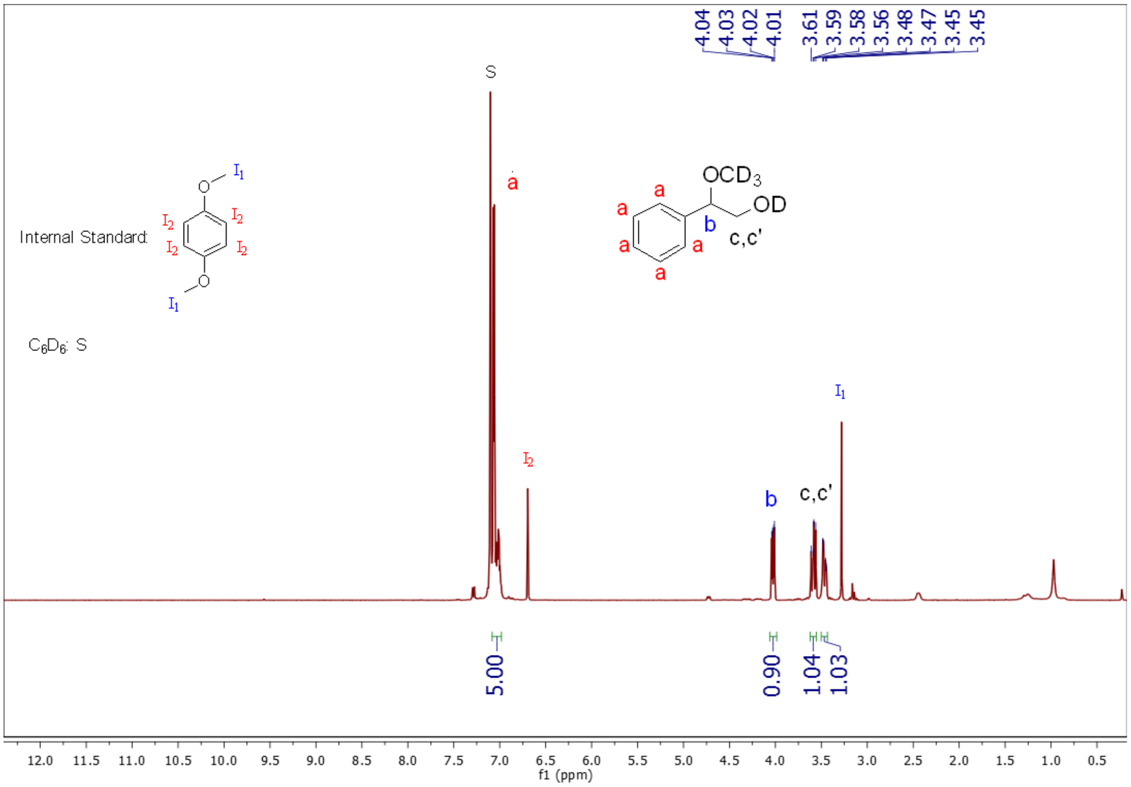

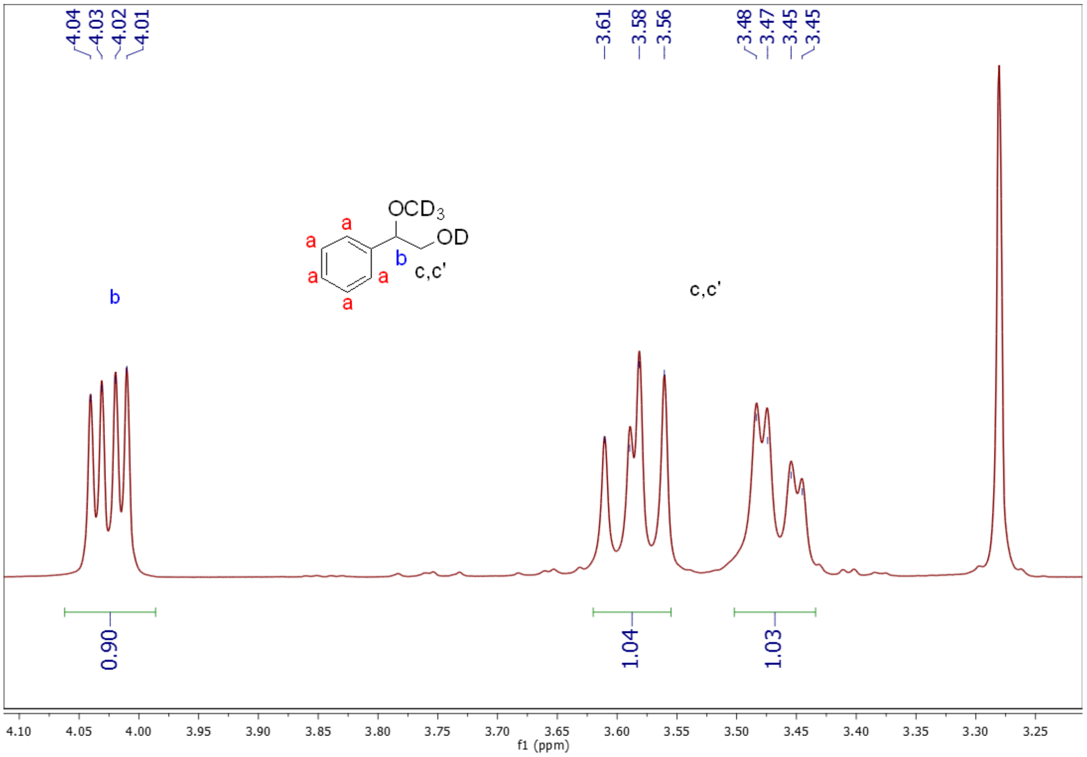

Figure S9 ^{1}H NMR (400 MHz, C_6D_6) spectrum of 2-methoxy$_{(d3)}$-2-phenylethan-1-ol$_{(d)}$.

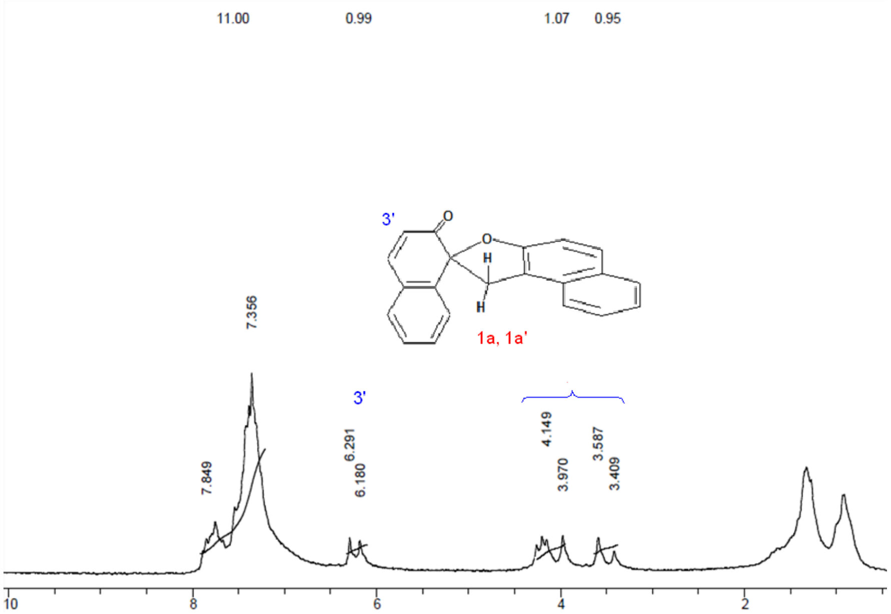

Figure S10 ^{1}H NMR (90 MHz, $CDCl_3$) spectrum of Spiro[naphthalene-1(2H),2′(1′H)-naphtho[2,1-b]furan]2-one.

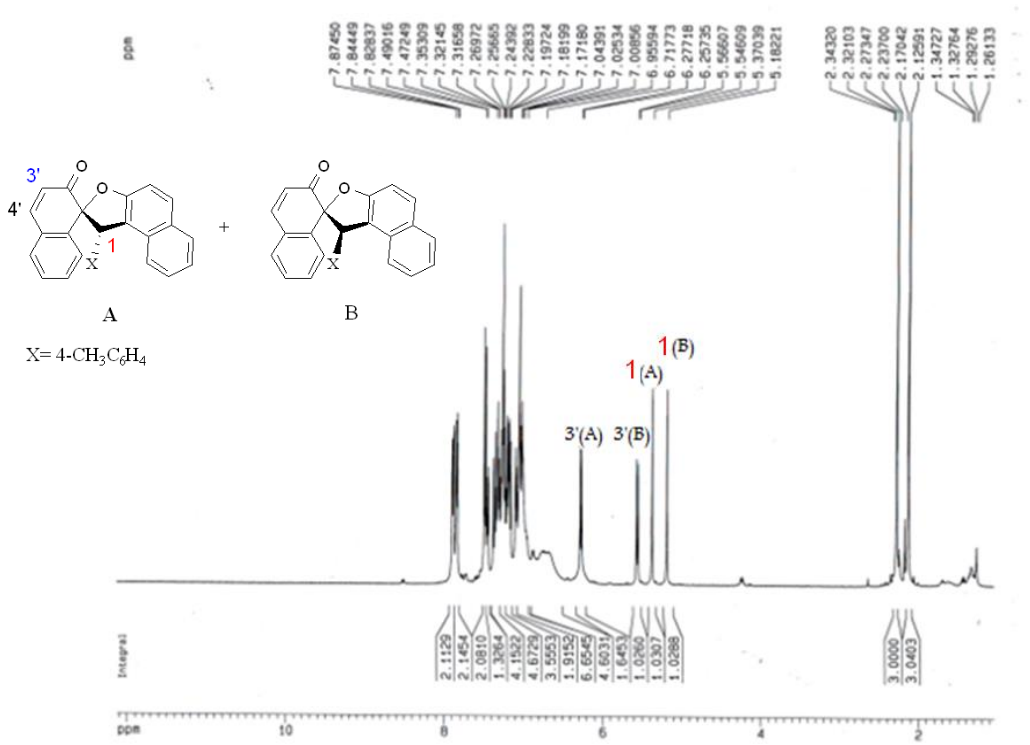

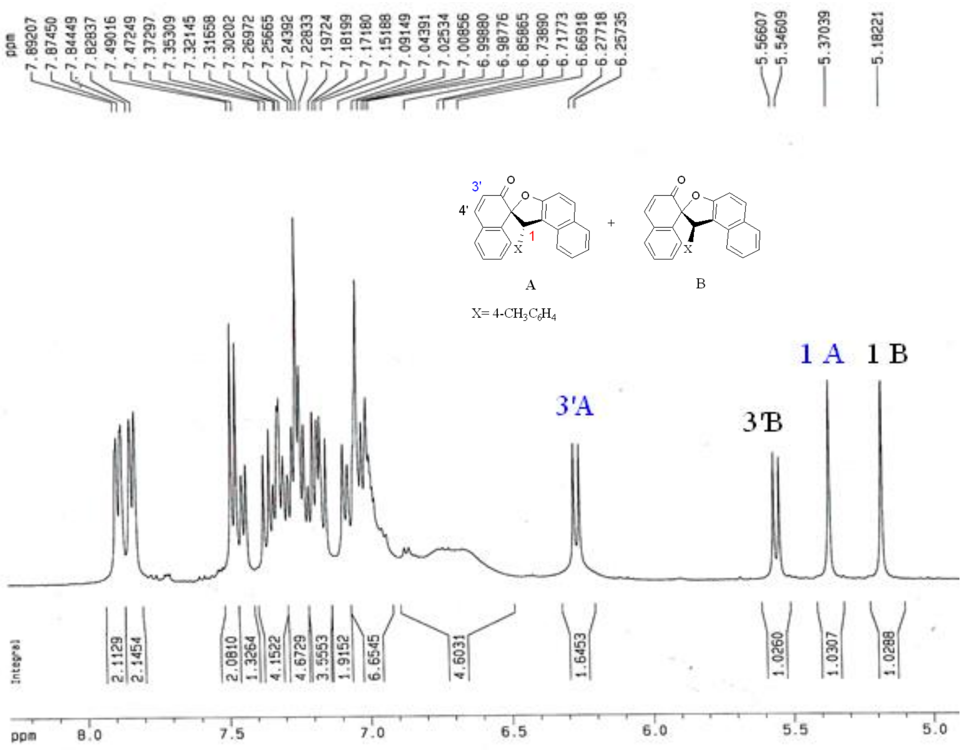

Figure S11 ^{1}H NMR (500 MHz, $CDCl_3$) spectrum of 1′-(4-Methylphenyl)-spiro[naphthalene-1(2H),2′(1′H)-naphtho[2,1-b]furan]-2-one.

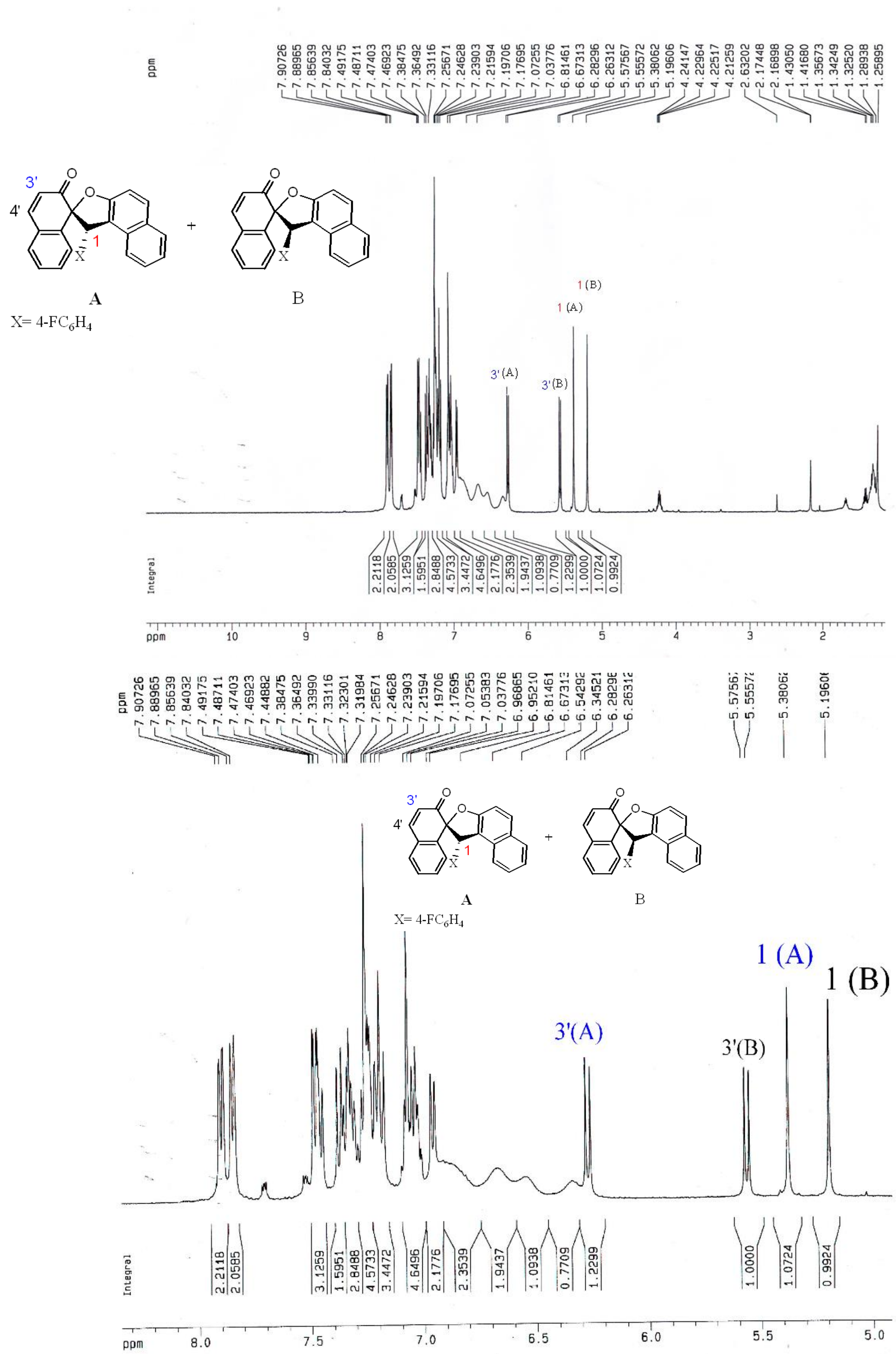

Figure S12 ^{1}H NMR (500 MHz, $CDCl_3$) spectrum of 1′-(4-Fluorolphenyl)-spiro[naphthalene-1(2H),2′(1′H)-naphtho[2,1-b]furan]-2-one.

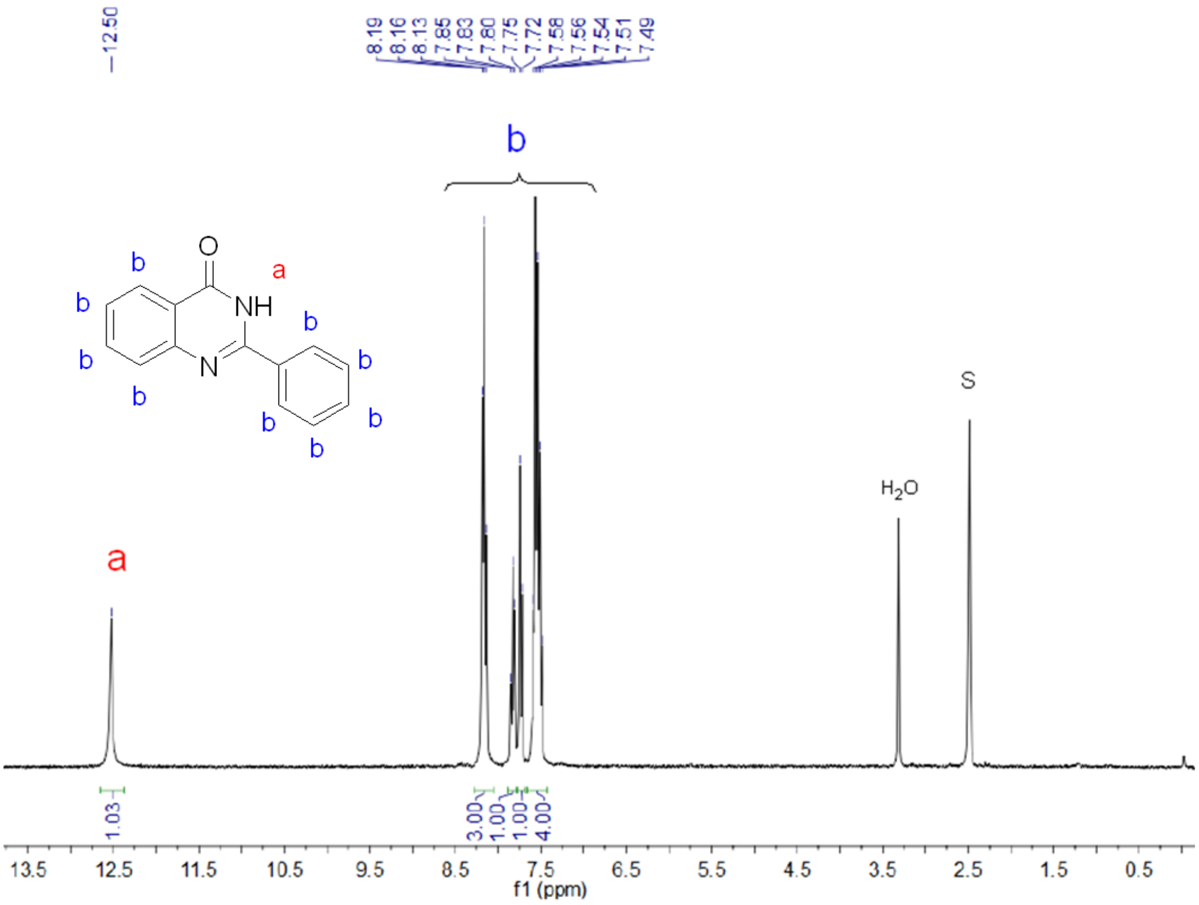

Figure S13 ^{1}H NMR (400 MHz, DMSO-d_6) spectrum of 2-phenyl-quinazoline-4(*3*H)-one.

S17. References

1. See this paper and its references: Khorramabadi-zad, A., Daliran, S., Oveisi, A. R. Aerial oxidation of bisnaphthols to spironaphthalenones by a recyclable magnetic core-sell nanoparticle-supported TEMPO catalyst. *C. R. Chim.* **16**, 972-976 (2013).

2. Lee, S. H., *et al.* Novel polymer-supported ruthenium and iron complexes that catalyze the conversion of epoxides into diols or diol mono-ethers: clean and recyclable catalysts. *New J. Chem.* **31**, 1579-1582 (2007).

3. Iranpoor, N., Tarrian, T., Movahedi, Z. $FeCl_3$•$6H_2O$ Supported on SiO_2 Catalysed Ring Opening of Epoxides with Alcohols, Acetic Acid, Water, Chloride, Bromide and Nitrate Ions. *Synthesis* **1996**, 1473-1476 (1996).

4. Dhakshinamoorthy, A., Alvaro, M., Garcia, H. Metal–Organic Frameworks as Efficient Heterogeneous Catalysts for the Regioselective Ring Opening of Epoxides. *Chem. Eur. J.* **16**, 8530-8536 (2010).

5. Beyzavi, M. H., *et al.* A Hafnium-Based Metal–Organic Framework as an Efficient and Multifunctional Catalyst for Facile CO_2 Fixation and Regioselective and Enantioretentive Epoxide Activation. *J. Am. Chem. Soc.* **136**, 15861-15864 (2014).

6. Khoramabadi-Zad, A., Yavari, I., Shiri, A., Bani, A. Oxidation of bisnaphthols to spironaphthalenones, revisited. *J. Heterocycl. Chem.* **45**, 1351-1358 (2008).

7. Ge, W., Zhu, X., Wei, Y. Iodine-catalyzed oxidative system for cyclization of primary alcohols with o-aminobenzamides to quinazolinones using DMSO as the oxidant in dimethyl carbonate. *RSC Adv.* **3**, 10817-10822 (2013).

8. Zhou, J., Fang, J. One-Pot Synthesis of Quinazolinones via Iridium-Catalyzed Hydrogen Transfers. *J. Org. Chem.* **76**, 7730-7736 (2011).

9. Watson, A. J. A., Maxwell, A. C., Williams, J. M. J. Ruthenium-catalysed oxidative synthesis of heterocycles from alcohols. *Org. Biomol. Chem.* **10**, 240-243 (2012).
